# HPLC-DAD-ESI-MS Analysis of Flavonoids from Leaves of Different Cultivars of Sweet Osmanthus

**DOI:** 10.3390/molecules21091224

**Published:** 2016-09-12

**Authors:** Yiguang Wang, Jianxin Fu, Chao Zhang, Hongbo Zhao

**Affiliations:** Department of Ornamental Horticulture, School of Landscape Architecture, Zhejiang Agriculture and Forestry University, Lin’an 311300, China; wangyiguang1990@163.com (Y.W.); fujianxin2008@sohu.com (J.F.)

**Keywords:** *Osmanthus fragrans* Lour., leaves, HPLC-DAD-ESI-MS, flavonoids, cultivar groups, chemotaxonomy

## Abstract

*Osmanthus fragrans* Lour. has traditionally been a popular ornamental plant in China. In this study, ethanol extracts of the leaves of four cultivar groups of *O. fragrans* were analyzed by high-performance liquid chromatography coupled with diode array detection (HPLC-DAD) and high-performance liquid chromatography with electrospray ionization and mass spectrometry (HPLC-ESI-MS). The results suggest that variation in flavonoids among *O. fragrans* cultivars is quantitative, rather than qualitative. Fifteen components were detected and separated, among which, the structures of 11 flavonoids and two coumarins were identified or tentatively identified. According to principal component analysis (PCA) and hierarchical cluster analysis (HCA) based on the abundance of these components (expressed as rutin equivalents), 22 selected cultivars were classified into four clusters. The seven cultivars from Cluster III (‘Xiaoye Sugui’, ‘Boye Jingui’, ‘Wuyi Dangui’, ‘Yingye Dangui’, ‘Danzhuang’, ‘Foding Zhu’, and ‘Tianxiang Taige’), which are enriched in rutin and total flavonoids, and ‘Sijigui’ from Cluster II which contained the highest amounts of kaempferol glycosides and apigenin 7-*O*-glucoside, could be selected as potential pharmaceutical resources. However, the chemotaxonomy in this paper does not correlate with the distribution of the existing cultivar groups, demonstrating that the distribution of flavonoids in *O. fragrans* leaves does not provide an effective means of classification for *O. fragrans* cultivars based on flower color.

## 1. Introduction

*Osmanthus fragrans* Lour. (Oleaceae), a small evergreen tree, is popular due to its aesthetic value and fragrance. As a traditional ornamental plant, *O. fragrans* has been cultivated for more than 2500 years in China. Exhibiting abundant variation, cultivars of *O. fragrans* have been classified into four groups, Albus Group, Luteus Group, Aurantiacus Group, and Asiaticus Group, based on flowering season and flower color [1]. Asiaticus Group cultivars, also called the four-season group, bloom in autumn and several other times during the entire year. In contrast, cultivars in the other three groups only bloom in autumn. These three autumn cultivar groups can be distinguished by flower color: cultivars in the Albus Group produce white to yellowish white flowers (RHSCC value of 1 to 8), those in the Luteus Group have yellow flowers (RHSCC value of 9–20), and flowers of the Aurantiacus Group are orange to orange-red in color (RHSCC value of 21–30).

In China, *O. fragrans* is regarded as both a source of edible products and traditional folk medicine as well as an ornamental plant. Due to its unique and strong fragrance, the flowers of *O. fragrans* are often used as a common additive for food, tea, and other beverages. In previous research, a large amount of antioxidant compounds, such as flavonoids and phenolics was detected in the flowers from this tree [2,3]. Additionally, the extract of *O. fragrans* flowers was found to possess pharmacological properties of anti-oxidative activity [4], free radical scavenging, neuroprotection [5], inhibitory effects on melanogenesis [6], and nitric oxide production [7]. Thus, *O. fragrans* is considered to be beneficial with regard to alleviating pain and relieving cough, rheumatism, and halitosis in Chinese folk medicine. Furthermore, the pulps and seeds of *O. fragrans* which are also traditional Chinese medicines, possess antioxidant activity, due to their unique phytochemical composition [8,9,10].

*O. fragrans* leaves, are evergreen and leathery and are rarely utilized well. Nonetheless, the leaves of *O. heterophyllus*, another *Osmanthus* species, have been used as an herbal drug for vitiligo vulgaris in Japan [11]. Indeed, previous studies have shown that the leaves of *Osmanthus* plants contain various medicinal components, such as phenolic glycosides, lignan glucosides phenylpropanoid glycosides, phenylethanoid glycosides, iridoid glycosides, and flavonoids [12,13,14,15,16,17,18,19,20]. Among these phytochemicals, flavonoids constitute one of the most widely studied class of food bioactive compounds, which exhibit many benefits for human health, including antioxidant [21], anti-inflammatory [22], antibacterial [23], and even anticancer [24,25] activities. Therefore, flavonoids are considered to be active ingredients of many medicinal plants, for example, *Polygonum hydropiper* Linn. [26], *Diospyros kaki* Thunb. (Polygonaceae) [27], *Ilex cornuta* Lindl. ex Paxt. (Aquifoliaceae) [28], and *Scutellaria baicalensis* Georgi (Labiatae) [29]. It has been reported that *O. fragrans* flowers contain a large amount of flavonoids (nearly 80 mg/g, DW), including rutin, isoquercitrin, quercitrin, and quercetin [2]. Flavonoid aglycones, such as quercetin, kaempferol, luteolin, and apigenin were also found in the leaves of *O. fragrans* Lour. var. aurantiacus Makino (this is an non-standard scientific name at present; the normal name is *O. fragrans* Aurantiacus Group) [16], suggesting that *O. fragrans* leaves also have potential value for utilization. However, there are few studies to date focusing on flavonoids in the leaves of *O. fragrans*, especially different cultivars.

In this study, high-performance liquid chromatography coupled with diode array detection (HPLC-DAD) and high-performance liquid chromatographic with electrospray ionization and mass spectrometry (HPLC-ESI-MS) were applied to identify and quantify flavonoids in the leaves of different *O. fragrans* cultivars. To select high quality germplasm resources with pharmaceutical potential, comparison of the contents of each flavonoid and total flavonoids in leaves of *O. fragrans* cultivars was performed. Furthermore, principal component analysis (PCA) and hierarchical cluster analysis (HCA) were used to classify the selected cultivars and to test for any relationships between flavonoid components and the cultivars or cultivar groups to provide information for chemotaxonomic studies.

## 2. Results

### 2.1. Qualitative Analysis of Leaf Flavonoids of O. fragrans

**Figure 1:**
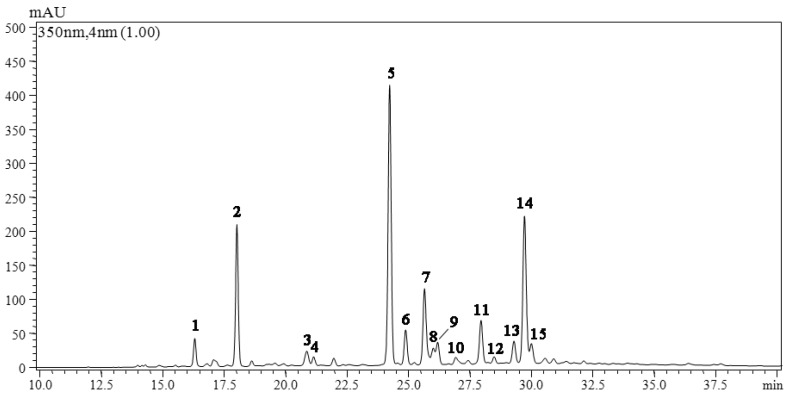
HPLC chromatogram of flavonoids extracts from the leaves of *O. fragrans.*

Fifteen constituents were found in ethanol-based extracts of all leaf samples at the characteristic wavelength of 350 nm (Figure 1). Among them, three flavonols (quercetin, kaempferol, and isorhamnetin), two flavone (luteolin and apigenin), one flavanone (naringenin), and two coumarins were identified or tentatively identified based on data of retention time, wavelength of maximum UV absorption, and mass spectrometry (MS) fragments in the positive mode corresponding to these peaks (Table 1).

Combined with the information of *m*/*z* 611 [M + H]^+^, *m*/*z* 465 [M + H−146]^+^, and *m*/*z* 303 [Y_0_^+^], and the wavelength of maximum UV absorption, peak five was deduced to be quercetin diglycoside linking one glucosyl and one rhamnosyl. The structure was confirmed as quercetin 3-*O*-rutinose (rutin) by co-elution with standard rutin. Peaks one, eight, and nine were also identified as quercetin derivatives based on the [Y_0_^+^] ion of *m*/*z* 303. Peak one showed mass spectrometric behaviors similar to those of peak five. The fragment ions and relative abundance indicated it is an isomer of rutin, quercetin 7-*O*-neohesperidoside. Hence, peaks eight and nine were identified as quercitrin 3-*O*-rhamnoside and quercitrin 3-*O*-glucoside using fragmentation data (loss of *m*/*z* 146 u and *m*/*z* 162 u, respectively).

Peaks six, seven, and 14 produced major fragments at *m*/*z* 287 [Y_0_^+^] in PI mode; thus, they were speculated to be luteolin glycosides or kaempferol glycosides. The UV spectrum of luteolin derivatives was approximately at 255 nm, whereas kaempferol derivatives were found at 265 nm [30]. Therefore, the aglycone of peak six was identified as luteolin; however, peaks seven and 14 were identified as kaempferol glycosides. According to MS data, peak six was deduced as luteolin 7-*O*-diglycosides. Furthermore, considering the relative abundances of protonated ions, Y_0_ > Y_1_ is consistent with a 1→2 linkage between the two monosaccharides [31]. Peak six was ultimately identified as luteolin 7-*O*-neohesperidoside. The loss of *m*/*z* 162, which is the molecule weight of hexose, revealed a hexose linked to the aglycone of peaks seven and 14. Since the UV-VIS spectrum of the 7-position showed a redshift compared with the 3-positon [32], peaks seven and 14 were identified as kaempferol 7-*O*-glucoside and kaempferol 3-*O*-glucoside, respectively.

Peaks 11 and 13 were identifed as apigenin derivatives due to the aglycone cation at *m*/*z* 271 and the characteristic UV spectrum. In a manner similar to peak six, peak 11 was assigned as apigenin 7-*O*-neohesperidoside. MS fragmentation data (loss of *m*/*z* 162 u) for peak 13 suggested it was linked with a hexose; thus, peak 13 was identified as apigenin 7-*O*-glucoside. Similarly, peak 12, which is the molecular weight of naringenin plus *m*/*z* 162 u was confirmed as naringenin 7-*O*-glucoside.

Peak two showed fragment ions at *m*/*z* 317, corresponding to isorhamnetin aglycone; this indicates that peak two was a type of isorhamnetin derivative. However, the structure could not be confirmed without more information. Owing to the low amount in the samples and the scant MS information, peaks three and four could not been identified. Regarding peaks 10 and 15, because the [Y_0_^+^] ions of *m*/*z* 209 correspond to the molecule weight of fraxetin, they were tentatively considered to be esculin (341 [M + H]^+^) and another fraxetin derivative, temporarily. Although these two constituents belong to the coumarin groups, they were analyzed with other flavonoid components in further research.

### 2.2. Component Analysis of Leaf Samples of O. fragrans

**Figure 2:**
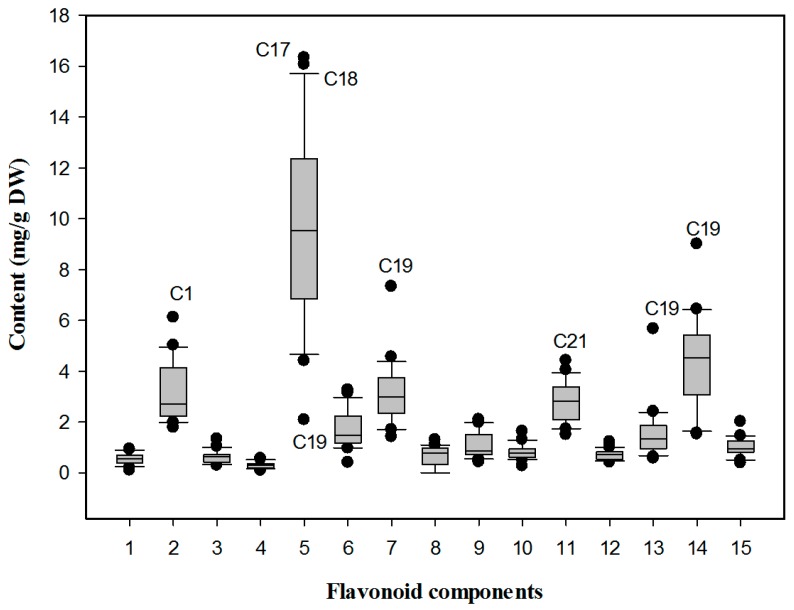
Box plot of flavonoid constitutions in leaf samples from different cultivars of *O. fragrans*. Component numbers are the same as in Figure 1, whereas sample numbers are the same as in Table 3.

The concentrations of each flavonoid and total flavonoids in the leaves of all samples, as determined by a semi-quantitative method using rutin as the standard at 350 nm, are shown in Table 2. Although quercitrin 3-*O*-rhamnoside (**8**) was not detected in C18, C19, C20, and C22, the categories of flavonoids were almost the same among the different samples. However, the content of flavonoid components showed significant variability among the 22 selected cultivars. An isorhamnetin derivative (**2**), quercetin 3-*O*-rutinose (**5**), kaempferol 7-*O*-glucoside (**7**), apigenin 7-*O*-neohesperidoside (**11**), and kaempferol 3-*O*-glucoside (**14**) were the dominant constituents in all cultivars (Figure 2). Among them, quercetin 3-*O*-rutinose (**5**) was present in the highest amounts in the leaf samples from most cultivars, accounting for 20.80% to 41.28% of the total flavonoids, except for C19. In addition, the highest value of quercetin 3-*O*-rutinose (16.33 mg/g, DW) was observed in C17, which also contained the most abundant total flavonoids (41.57 mg/g, DW). The contents of other components in the different cultivars also varied. The isorhamnetin derivative (**2**) content of C1 which contained the highest amount, was 2.43 times higher than that of C14. The apigenin 7-*O*-neohesperidoside (**11**) content of C21 was 1.95 times higher than that of C10. The kaempferol 7-*O*-glucoside (**7**), apigenin 3-*O*-glucoside (**13**) and kaempferol 3-*O*-glucoside (**14**) contents of C19 were detected at the highest levels among these cultivars. The levels of these components in the leaves of C19 were 4.1, 9.1, and 4.9 times higher than those of C4, C21, and C16, the cultivars that contained the lowest amounts, respectively. These results suggested that variation in flavonoids among *O. fragrans* cultivars is quantitative rather than qualitative.

### 2.3. Cluster Analysis of Different Cultivars

**Figure 3:**
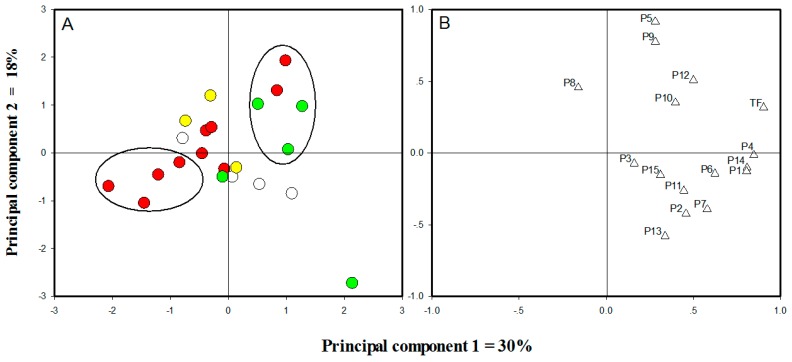
Principal component analysis of *O. fragrans* cultivars based on PC1 and PC2 scores. (**A**) Score plot of cases: white dots denote cultivars from Albus Group, yellow dots denote cultivars from Luteus Group, red dots denote cultivars from Aurantiacus Group, and green dots denote cultivars from Asiaticus Group; (**B**) Score plot of variables: triangles denote components from *O. fragrans* leaves, for which numbers are the same as in Figure 1.

To classify the selected cultivars, quantitative data of flavonoids in leaf samples from different cultivars were subjected to principal components analysis (PCA) using IBM SPSS Statistics 19.0 (IBM, Armonk, NY, USA). The *O. fragrans* cultivars were located at different points in the two-dimensional space described by two PCs, principal component 1 (30%) and principal component 2 (18%) (Figure 3A). The result revealed that these cultivars could be marginally distinguished, according to their locations in different quadrants. PC1 was positively correlated with most flavonoid components, especially the total flavonoids (TF), whereas PC2 showed a significant positive correlation with quercetin 3-*O*-rutinose (**5**) and quercitrin 3-*O*-glucoside (**9**), and a markedly negative correlation with apigenin 7-*O*-glucoside (**13**) (Figure 3B). Thus, the cultivars (two Aurantiacus Group and three from Asiaticus Group) clustering at positive PC1 and positive PC2 were characterized by the highest amounts of total flavonoids and rutin. In contrast, four cultivars from Aurantiacus Group were located at the quadrant of negative PC1 and negative PC2; these were characterized by the lowest flavonoid level in the dataset. Cultivars from Aurantiacus Group, which were rarely placed in the quadrant left along PC1 and above along PC2, were correlated with the absence of quercitrin 3-*O*-rhamnoside (**8**). It is worth noting that due to high amounts of total flavonoids and apigenin 7-*O*-glucoside (**13**) and extremely low levels of rutin, the location of C19 was far from the other cultivars.

**Figure 4:**
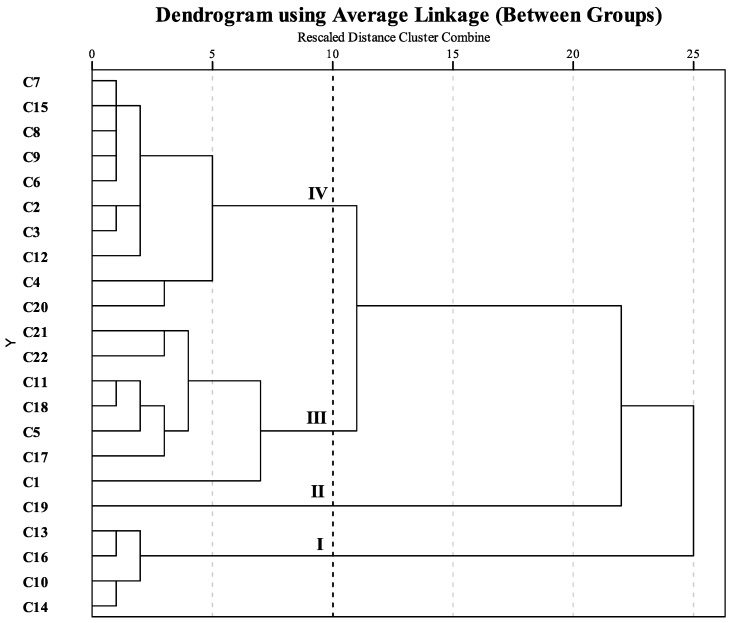
Dendrogram of hierarchical cluster analysis of leaf samples from 22 cultivars of *O. fragrans*. The abscissa indicates the Euclidean distances and the ordinate expresses the sample numbers. Sample numbers are the same as in Table 3.

In addition, hierarchical cluster analysis (HCA) was performed to obtain a specific classification of the 22 cultivars on the basis of 15 components and total flavonoids. Differences between samples are represented by Euclidean distances. Figure 4 shows a dendrogram of hierarchical cluster analysis of the 22 samples using the between-group linkage method. The abscissa expresses Euclidean distances, whereas the ordinate indicates sample numbers. As a result, which was similar to that of the PCA analysis, the samples were divided into four clusters when the Euclidean distances of the dendrogram was 10. Cluster I incorporated C13, C16, C10, and C14 which belonged to the Aurantiacus Group. Only C19 was separated as cluster II. Cluster III contained C1 of the Albus Group, C5 of the Luteus Group, C11 and C17 of the Aurantiacus Group, and C18, C21, and C22 of the Asiaticus Group. Cluster IV contained C2, C3, C4 of the Albus Group, C6 and C7 of the Luteus Group, C8, C9, C12, and C15 of the Aurantiacus Group, and C20 of the Asiaticus Group.

**Figure 5:**
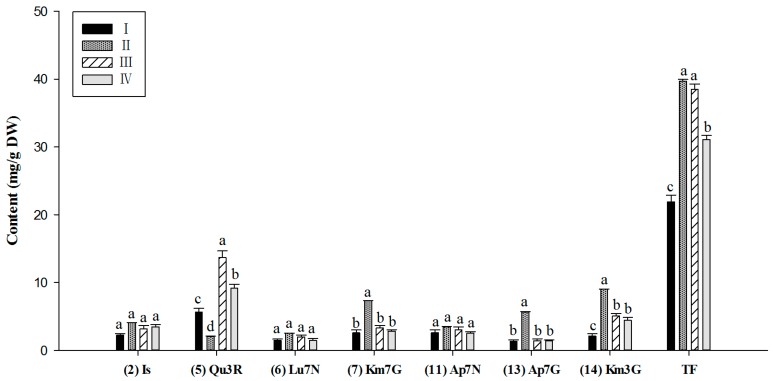
Dominant flavonoid components of the four clusters. The data are the means ± SE of measurements from cultivars belonging to each cluster. Significant differences (*p* ≤ 0.05 by Least Significant Difference test) in flavonoid content between the different clusters are indicated by a, b, and c. Component numbers are the same as in Figure 1. TF: total flavonoids.

Based on the analysis of variance for the major flavonoid components and total flavonoids of these four clusters, the levels could be distinguished (Figure 5). Significant differences in quercetin 3-*O*-rutinose (**5**), kaempferol 7-*O*-glucoside (**7**), kaempferol 3-*O*-glucoside (**14**), apigenin 7-*O*-glucoside (**13**), and total flavonoid contents were found, whereas the contents of isorhamnetin derivative (**2**), luteolin 7-*O*-neohesperidoside (**6**), and apigenin 7-*O*-neohesperidoside (**11**) were similar in the different clusters. Cluster I contained the lowest concentration of total flavonoids (21.90 ± 1.02 mg/g, DW), and the levels of dominant components were also lower than in other clusters. Conversely, the leaves of cluster III cultivars accumulated the highest levels of rutin (13.79 ± 0.98 mg/g, DW) and total flavonoids (38.50 ± 0.80 mg/g, DW). Of note, the leaf sample of cluster II cultivar was characterized by a relatively higher content of kaempferol glycosides, apigenin 7-*O*-glucoside and total flavonoids, whereas the quercetin 3-*O*-rutinose (**5**) level was significantly lower than in other clusters. The remaining cultivars were grouped as the fourth cluster, which contained moderate level of total flavonoids.

## 3. Discussion

**Figure 6:**
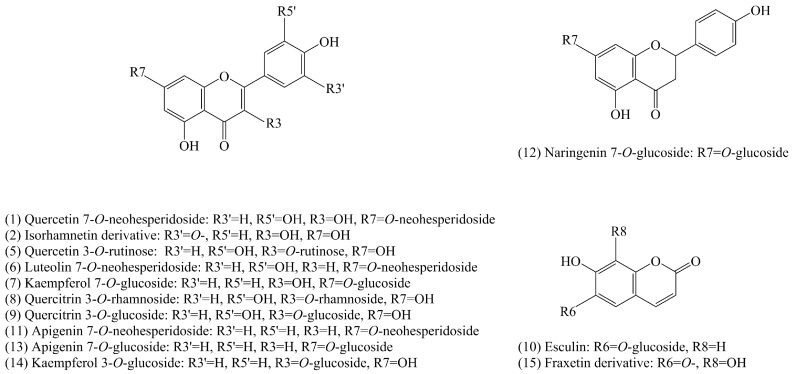
Chemical structures of tentatively-identified components from *O. fragrans* leaves. Component numbers are the same as in Figure 1.

Although the leaf chemical constituents of several *Osmanthus* species have been researched previously [12,13,14,15,16,17,18,19,20], these reports appear to pay more attention to total chemical constituents as opposed to flavonoids. In our present study, we focused on the analysis of flavonoid components in the leaves of *O. fragrans*. Fifteen components from *O. fragrans* leaves were detected, among them, the structures of 11 flavonoids and two coumarins were tentatively identified (Figure 6). To our knowledge, two components were detected for the first time and deduced as esculin and fraxetin derivatives, respectively. These two phytochemicals are of the coumarin groups, precursors of flavonoids, were largely found in *Fraxinus ornus* Linn. (Oleaceae) [33] and *Aesculus hippocastanum* Linn. (Hippocastanaceae) [34], and are considered to be valuable medicinal constitutents with spasmolytic and diuretic properties. However, due to the low concentration, these two components could only be tentatively identified. More information is needed to confirm their unambiguous structures in the future.

**Figure 7:**
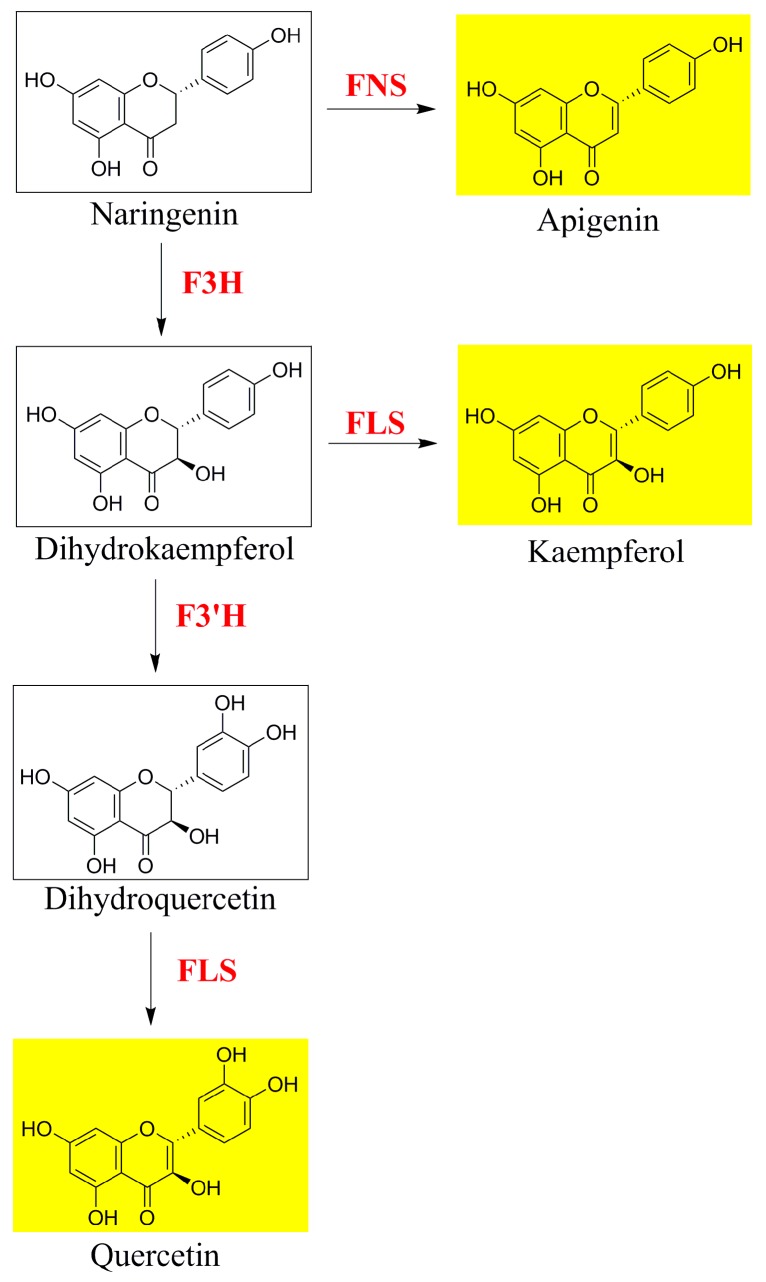
Local diagram of the flavonoid biosynthetic pathway. FNS: flavone synthase; F3H: flavanone 3-hydroxylase; FLS: flavonol synthase; and F3′H: flavonoid 3′-hydroxylase

According to the quantitative analysis, we found that although the amount of flavonoids in *O. fragrans* leaves (19.80–41.57 mg/g, DW) was much lower than that in *O. fragrans* flowers [2,6], the levels were still on par with those of the leaves of lotus (*Nelumbo nucifera* Gaertn) [35], which is also used in traditional Chinese medicine. Moreover, quercetin 3-*O*-rutinose, known as rutin, which was reported as a phenolic antioxidant in *O. fragrans* flowers [2,3], was found to be the dominant flavonoid in leaves. The cultivars in cluster III (seven cultivars, ‘Xiaoye Sugui’, ‘Boye Jingui’, ‘Wuyi Dangui’, ‘Yingye Dangui’, ‘Danzhuang’, ‘Foding Zhu’, and ‘Tianxiang Taige’), especially ‘Yingye Dangui’ with the highest amounts of rutin, are potential candidates for selecting appropriate medicinal resources rich in rutin and total flavonoids. As the second most abundant flavonol aglycone in *O. fragrans* leaves, kaempferol, also found in many fruits and vegetables, has beneficial effects in reducing the risk of chronic diseases, especially cancer [36]. *O. fragrans* ‘Sijigui’, a representative cultivar of the Asiaticus Group, accumulated the highest level of kaempferol among the selected 22 cultivars. This cultivar could be a resource for extraction of apigenin 7-*O*-glucoside due to the much higher amount found compared to the other cultivars. In the flavonoid biosynthetic pathway of seed plants (Figure 7), naringenin is catalyzed by flavone synthase (FNS) to form apigenin, and it can also be hydroxylated at the 3-position by flavanone 3-hydroxylase (F3H) to yield dihydrokaempferol. In the next step, catalyzed by flavonol synthase (FLS) and flavonoid 3′-hydroxylase (F3′H), dihydrokaempferol is converted to kaempferol and dihydroquercetin, the precursor of quercetin, respectively [37]. Accordingly, we speculate the existence of a block in the metabolic pathway to quercetin, which most likely explains the higher kaempferol and apigenin levels and notably lower quercetin contents of ‘Sijigui’ leaves compared with the other cultivars. In contrast, the flavonoid biosynthetic pathway in the cultivars of cluster III appears to predominantly lead to quercetin.

Since flavonoids are used as effective chemotaxonomic markers in the classification of many angiosperm plants [38], Harborne and Green [39] conducted a comprehensive chemotaxonomic survey of flavonoids in the family Oleaceae and approximately divided those species and genera into two groups based on flavonol glycosides alone, or both flavonol and flavone glycosides, which correlated with chromosome number (and subfamily division). More recently, work by Lee et al. [40] showed that a flavonoids survey of *Fraxinus* (Oleaceae) was useful for determining natural groupings of several overlooked species. In the present work, the data obtained failed to clearly distinguish each groups of *O. fragrans* cultivars due to the similar flavonoid compositions. Nonetheless, quantitative analysis revealed that each *O. fragrans* cultivar has the distinctive ability to accumulate flavonoids. Hence, principal components analysis (PCA) and hierarchical cluster analysis (HCA) based on the amounts of flavonoid components were used to classify the selected cultivars, dividing them into four clusters. Disappointingly, the result was still not be in accordance with the classification of cultivar groups based on flower color. Indeed, most cultivars of each cultivar group were scattered across the four clusters, with only the four cultivars of the Aurantiacus Group clustered together in cluster I due to their lowest flavonoids levels. The flower colors of these four cultivars are orange to orange-red, and three of them, ‘Zhuangyuan Hong’ (RHSCC N25A), ‘Chenghong Dangui’ (RHSCC N25A-25B) and ‘Mantiao Hong’ (RHSCC 28A), display a remarkably redder flower color than the other cultivars tested [1,41]. However, a previous report [42] indicated that flower color of *O. fragrans* is mainly determined by carotenoids which are produced via a metabolic pathway that is quite different from that of flavonoids. To our knowledge, morphological characteristics are important basics of cultivar classification. As an obvious ornamental characteristic, flower color has been used for the taxonomy of cultivar groups in *O. fragrans*, even though epigenetic changes, such as flower color, may not always reflect phylogenetic evolution. Using molecular markers, it was revealed that the genetic relationships among the four cultivar groups of *O. fragrans* are also inconsistent with the classification based on flower color [43]. Taking together these findings, it is reasonable to presume that there are no relationships linking leaf flavonoids with the artificial classification of *O. fragrans* cultivar groups. Regardless, more studies on the metabolic pathways of flavonoids and carotenoids in different *O. fragrans* cultivars are needed to investigate the diversity of *O. fragrans* cultivar groups.

In conclusion, several cultivars and clusters were selected as potential pharmaceutical resources based on the specific flavonoid components detected in this work. Principal components analysis (PCA) and hierarchical cluster analysis (HCA) based on flavonoids in the leaves of different *O. fragrans* cultivars could not offer a good approach for chemotaxonomy, perhaps taxonomy based on phytochemicals is more effective for categorizing at the levels lower than species or genus.

## 4. Materials and Methods

### 4.1. Plant Materials

Studies were conducted on 22 cultivars, belonging to four cultivar groups of *O. fragrans* (Table 3). The plant materials used in this study were cultivated under the same condition at the germplasm repository for *O. fragrans* (located at 30°15’23’’ N/ 119°43’37’’ E), Zhejiang Agriculture and Forestry University, Lin’an, China. Leaf samples were obtained from three plants of each cultivar, and five mature leaves were collected from four growth directions of every plant in July 2015. The samples were dried using allochroic silica gel and stored at −80 °C for later analysis.

### 4.2. Chemicals and Reagents

Quercetin 3-*O*-rutinoside (rutin) was purchased from Maya reagent (Jiaxing, Zhejiang, China); the purity of the standard was above 98%. HPLC-grade methanol, acetonitrile, and formic acid were purchased from DikmaPure (Lake Forest, CA, USA). Analytical-grade ethanol was obtained from Sinopharm Chemical Reagent (Shanghai, China). Water for liquid chromatography was prepared using a Milli-Q water purification system (Millipore, Bedford, MA, USA).

### 4.3. Extraction of Flavonoids

The dried leaf samples of each cultivar from different sampling points and trees were ground into powder and mixed together adequately. One gram of each sample was mixed with 50 mL of 70% (*v*/*v*) ethanol, and then placed on a rotating shaker at 200 rpm and room temperature in the dark for 16 h. All samples were extracted in triplicate. The extracts were filtered through a 0.22 μm nylon filter prior to HPLC and HPLC-MS analysis.

### 4.4. Quantitative Determination of Flavonoids

Flavonoids were quantitatively determined using a Shimadzu HPLC system (Kyoto, Japan), consisting of an LC-20AT pump, a SPD-M20A DAD detector, a CTO-10AS VP column oven, and a SIL-20A auto injector. A 10 μL aliquot of each sample was eluted through a C18 column of Inertsil ODS SP (4.6 mm × 250 mm, 5 μm) at a column temperature of 28 °C and a flow rate of 0.8 mL/min. The mobile phase consisted of solvent A (0.1% formic acid) and B (acetonitrile). The gradient elution programs used was as follows: 0–5 min, 5%–15% B; 5–25 min, 15%–30% B; 25–39 min, 30%–38% B; 39–47 min, 38%–55% B; 47–50 min, 55%–70% B; 50–56 min, 70%–75% B; 56–60 min, 75%–100% B; 60–65 min, 100%–5% B; 65–75 min, 5% B. Chromatograms were obtained at 350 nm, and UV diode array detection spectra were scanned from 190–800 nm.

The contents of total flavonoids and individual flavonoids in different samples were measured using a semi-quantitative method based on a linear regression of rutin at 350 nm, as calculated using mg per 1 g dry leaves. Each single sample was analyzed in triplicate. The calibration curve was constructed by plotting the peak areas versus the different concentrations of the working standard solution in triplicate. The regression equation was: *y* = 19727*x* − 3899 (*r*^2^ = 0.9999). The limit of detection (LOD) and limit of quantitation (LOQ) were 0.062 μg/mL and 0.189 μg/mL, respectively.

### 4.5. Identification of Flavonoids

Each flavonoid in *O. fragrans* was identified by HPLC-ESI-MS using a Thermo Ion trap mass spectrometer (Waltham, MA, USA) equipped with an electrospray ionization (ESI) interface. The ESI-MS was applied in positive modes, and the product ions from protonated molecular ions were further analyzed by MS/MS (MS^2^). Nitrogen was the drying gas and nebulizing gas. Other parameters consisted of a drying gas temperature of 350 °C, capillary voltage of 3.5 kV, capillary exit voltage of 120.4 V, cap exit offset voltage of 77.2 V, and range of *m*/*z* 100–1000 for full scan MS analysis.

### 4.6. Statistical Analysis

The contents of each flavonoids and total flavonoids were expressed as the means calculated using three replicate analysis of triplicate extraction. Principal components analysis (PCA) and hierarchical cluster analysis (HCA) with the between-group linkage method were performed using IBM SPSS Statistics 19.0 (IBM, Armonk, NY, USA). The variance of flavonoid content in the leaves of cultivars from different clusters was analyzed by Least Significant Difference (LSD) test. Significant differences was determined at *p* ≤ 0.05.

## Figures and Tables

**Table 1:** HPLC-ESI-MS^2^ analysis and tentative identification of flavonoids in the leaves of *O. fragrans.*

Peaks ^a^	*t*_R_ (min)	λmax (nm)	ESI-PI MS/MS^2^ (*m*/*z*)	Tentative Identification
1	16.26	272, 326	611.1[M + H]^+^, 427.1, 303[Y_0_^+^]	Quercetin 7-*O*-neohesperidoside
2	17.93	270, 334	595.1[M + H]^+^, 457.1, 385.1, 317.0[Y_0_^+^]	Isorhamnetin derivative
3	20.78	238, 325	564.3[M + H]^+^, 410.1	No tentative identification
4	20.99	289, 332	449.1[M + H]^+^, 265.0, 211.0	No tentative identification
5	24.02	252, 334	611.1[M + H]^+^, 465.0, 303[Y_0_^+^]	Quercetin 3-*O*-rutinose
6	24.66	253, 345	595.1[M + H]^+^, 448.8 (82) [Y_1_^+^], 433 (4), 287.1[Y_0_^+^] (100)	Luteolin 7-*O*-neohesperidoside
7	25.43	266, 345	448.8[M + H]^+^, 287.1[Y_0_^+^]	Kaempferol 7-*O*-glucoside
8	25.80	284, 330	449.1[M + H]^+^, 303[Y_0_^+^]	Quercitrin 3-*O*-rhamnoside
9	25.94	281, 329	465.0[M + H]^+^, 303.1[Y_0_^+^]	Quercitrin 3-*O*-glucoside
10	26.68	265, 332	341.1[M + H]^+^, 209.0[Y_0_^+^]	Esculin
11	27.69	266, 334	578.9[M + H]^+^, 432.8(65) [Y_1_^+^], 271.1[Y_0_^+^](100)	Apigenin 7-*O*-neohesperidoside
12	28.23	284, 332	435.2[M + H]^+^, 273.2[Y_0_^+^]	Naringenin 7-*O*-glucoside
13	29.04	267, 333	432.8[M + H]^+^, 271.1[Y_0_^+^]	Apigenin 7-*O*-glucoside
14	29.46	268, 335	448.9[M + H]^+^, 287.1[Y_0_^+^]	Kaempferol 3-*O*-glucoside
15	29.74	268, 324	435.1[M + H]^+^, 322.1, 209.0[Y_0_^+^]	Fraxetin derivative

^a^ Component numbers are the same as in Figure 1.

**Table 2:** Contents (mg/g, DW) expressed as rutin equivalents of 15 compounds and total flavonoids in leaf samples from 22 cultivars of *O. fragrans*.

Sample ^a^ No.	Contents (mg/g, DW)															
	1 ^b^	2	3	4	5	6	7	8	9	10	11	12	13	14	15	TF
C1	0.59	6.11	1.03	0.33	9.04	1.96	3.25	0.84	0.77	1.03	4.06	0.67	2.33	5.73	2.01	39.75
C2	0.71	4.25	0.63	0.36	7.94	2.37	2.86	0.45	0.76	0.82	3.25	0.58	0.91	6.40	1.24	33.52
C3	0.55	5.02	0.51	0.26	8.02	1.43	3.30	0.94	0.77	0.81	2.98	0.70	1.78	4.53	1.35	32.93
C4	0.49	4.20	0.98	0.23	9.54	0.40	1.42	0.46	1.85	0.84	2.87	0.52	1.17	1.79	1.16	27.91
C5	0.35	2.29	0.46	0.19	14.86	1.06	2.75	1.10	1.96	0.55	1.93	0.81	2.42	4.18	1.45	36.01
C6	0.85	4.11	0.76	0.39	9.70	1.32	2.99	0.75	0.92	0.63	1.89	0.46	1.09	5.70	0.99	32.54
C7	0.39	2.04	0.32	0.22	12.27	0.97	2.35	1.30	0.82	0.68	2.23	0.72	1.34	4.38	1.17	31.19
C8	0.38	2.25	0.73	0.17	9.67	1.38	3.96	1.07	0.74	1.12	1.77	0.52	2.14	5.34	0.95	32.18
C9	0.36	2.49	0.35	0.19	9.53	1.14	2.98	0.75	1.51	0.85	2.13	0.81	1.81	5.05	1.44	30.89
C10	0.33	2.44	0.59	0.16	4.41	1.18	3.88	n.d.	0.42	0.77	1.50	0.53	1.77	2.15	0.48	20.60
C11	0.77	2.67	0.53	0.33	14.74	1.48	4.56	0.88	1.54	1.63	2.24	1.00	2.00	3.85	0.94	39.15
C12	0.58	3.26	0.67	0.23	8.09	3.16	3.55	1.01	0.77	0.73	3.37	0.73	1.70	2.72	0.56	31.12
C13	0.20	1.97	0.72	0.18	5.85	1.54	2.23	0.88	0.58	0.51	3.25	0.67	1.53	3.18	0.88	24.18
C14	0.10	1.79	0.65	0.09	5.25	1.02	1.77	0.79	0.64	0.27	3.25	0.41	1.35	1.56	0.87	19.80
C15	0.54	2.32	0.66	0.36	11.22	1.25	2.74	0.72	1.13	1.19	1.73	0.53	0.99	4.52	0.97	30.85
C16	0.68	2.74	0.29	0.31	7.16	2.09	2.48	0.53	0.52	0.56	2.35	0.82	0.63	1.52	0.39	23.05
C17	0.59	2.09	0.43	0.35	16.33	2.20	3.87	1.10	1.97	0.91	2.80	1.22	0.82	6.44	0.82	41.57
C18	0.60	2.17	1.33	0.44	16.06	1.48	3.28	n.d.	2.09	0.53	2.32	0.75	0.97	5.33	0.67	38.01
C19	0.93	4.12	0.68	0.53	2.08	2.53	7.34	n.d.	0.62	0.63	3.49	0.71	5.67	9.00	1.35	39.72
C20	0.45	4.80	0.42	0.30	5.80	1.63	2.33	n.d.	0.91	0.88	3.46	0.96	1.04	3.94	0.98	27.88
C21	0.66	4.03	0.80	0.50	12.66	3.27	1.69	0.97	1.48	1.31	4.43	0.93	0.56	5.24	0.87	39.38
C22	0.91	2.74	0.36	0.56	12.19	2.48	3.71	n.d.	1.17	0.61	3.69	0.86	0.80	4.76	0.78	35.62

^a^ Sample numbers are the same as in Table 3. ^b^ Component numbers are the same as in Figure 1; TF denotes ‘total favonoids’; n.d. denotes ‘not detected’.

**Table 3:** Twenty-two *O. fragrance* cultivars of four cultivar groups used in this study.

Cultivar Groups	Sample No.	Cultivars
Albus Group	C1	*O. fragrans* ‘Xiaoye Sugui’
	C2	*O. fragrans* ‘Zaoyin Gui’
	C3	*O. fragrans* ‘Yu Linglong’
	C4	*O. fragrans* ‘Ziyin Gui’
Luteus Group	C5	*O. fragrans* ‘Boye Jingui’
	C6	*O. fragrans* ‘Hangzhou Huang’
	C7	*O. fragrans* ‘Jin Qiu’
Aurantiacus Group	C8	*O. fragrans* ‘Ruanye Dangui’
	C9	*O. fragrans* ‘Chilian Jindan’
	C10	*O. fragrans* ‘Mantiao Hong’
	C11	*O. fragrans* ‘Wuyi Dangui’
	C12	*O. fragrans* ‘Xionghuang’
	C13	*O. fragrans* ‘Zhuangyuan Hong’
	C14	*O. fragrans* ‘Chenghong Dangui’
	C15	*O. fragrans* ‘Zhusha Dangui’
	C16	*O. fragrans* ‘Yan Hong’
	C17	*O. fragrans* ‘Yingye Dangui’
Asiaticus Group	C18	*O. fragrans* ‘Danzhuang’
	C19	*O. fragrans* ‘Sijigui’
	C20	*O. fragrans* ‘Tiannu Sanhua’
	C21	*O. fragrans* ‘Foding Zhu’
	C22	*O. fragrans* ‘Tianxiang Taige’
